# Role of New Functional MRI Techniques in the Diagnosis, Staging, and Followup of Gynecological Cancer: Comparison with PET-CT

**DOI:** 10.1155/2012/219546

**Published:** 2012-01-18

**Authors:** Elena Alvarez Moreno, Mar Jimenez de la Peña, Raquel Cano Alonso

**Affiliations:** Department of Diagnostic Imaging, Hospital Quiron Madrid, Diego de Velázquez 1, 28223 Madrid, Spain

## Abstract

Recent developments in diagnostic imaging techniques have magnified the role and potential of both MRI and PET-CT in female pelvic imaging. This article reviews the techniques and clinical applications of new functional MRI (fMRI) including diffusion-weighted MRI (DWI), dynamic contrast-enhanced (DCE)-MRI, comparing with PET-CT. These new emerging provide not only anatomic but also functional imaging, allowing detection of small volumes of active tumor at diagnosis and early disease relapse, which may not result in detectable morphological changes at conventional imaging. This information is useful in distinguishing between recurrent/residual tumor and post-treatment changes and assessing treatment response, with a clear impact on patient management. Both PET-CT and now fMRI have proved to be very valuable tools for evaluation of gynecologic tumors. Most papers try to compare these techniques, but in our experience both are complementary in management of these patients. Meanwhile PET-CT is superior in diagnosis of ganglionar disease; fMRI presents higher accuracy in local preoperative staging. Both techniques can be used as biomarkers of tumor response and present high accuracy in diagnosis of local recurrence and peritoneal dissemination, with complementary roles depending on histological type, anatomic location and tumoral volume.

## 1. Introduction

The principal aim of oncological imaging is to differentiate between malignant and nonmalignant tissues at all stages of the patient's cancer care. Accurate staging and precise delineation of the extent of malignancy influence therapeutic decisions, therapy outcomes, and, ultimately, patient prognosis.

Conventional imaging using ultrasound, computed tomography (CT), or magnetic resonance imaging (MRI) detects cancer by identifying anatomical distortion or altered tissue appearances. Tumor tissue conspicuity may be increased after the administration of intravenous contrast medium, thus enhancing detection and delineation. However, identification of small volume active tumor, either at presentation or at early disease relapse remains challenging because a small volume of disease may not result in detectable structural or morphological changes on conventional imaging. Furthermore, the effects of therapy may obscure or mimic recurrent disease.

The use of functional imaging techniques using MRI and positron emission tomography (PET) in the evaluation of tumors is increasing. These techniques exploit particular pathophysiological changes occurring within tumors as their contrast mechanism, such as altered blood flow, increased glucose metabolism, and cellularity. Therefore, functional techniques are increasingly being used for tumor detection, monitoring of treatment response, and detection of relapsed disease.

## 2. PET-CT

PET imaging for oncology has tremendously advanced over the past 10–15 years. The most commonly employed radioisotope for PET imaging is 18-fluorodeoxyglucose (FDG). FDG is a glucose analogue and is injected into the patient, then transported across the cell membrane of actively metabolizing cells. Tumors that are visualized by FDG-PET imaging have glucose metabolic rates that are greater than surrounding normal cells. The standardised uptake value (SUV) represents a semiquantitative assessment of uptake in a tumor region of interest.

FDG is not, however, entirely specific for malignant cells, and there are a number of pitfalls when using this radiotracer. On the one hand, physiological uptake occurs in metabolically active normal tissues (i.e., brain, bowel, genitourinary tract, salivary glands, etc.). In premenopausal women, the uterus will often demonstrate FDG uptake most commonly during the menstrual flow phase [1]. Also in premenopausal women an ovarian uptake of FDG can be detected, which may be due to normal physiologic uptake or due to malignancy. In postmenopausal women, however, increased ovarian FDG uptake is highly associated with malignant processes. The urinary tract and urinary bladder will demonstrate FDG activity because FDG is eliminated in the urine and FDG bladder activity can obscure pelvic findings in these patients. Also focal areas of ureteral activity can mimic nodal disease. On the other hand, benign processes such as endometriosis, leiomyomata, inflammation, and infection can all be the causes of nonmalignant FDG uptake. Reactive lymph nodes can also be FDG avid. False negatives can also occur, as adenocarcinomas that have a low FDG uptake and may not be detected [2] and necrotic lymph nodes may not be FDG avid. FDG-PET/CT is limited in its ability to identify lesions <1 cm, in particular, those smaller than 5 mm, leading to a false-negative rate of 5%–10% [3]. Similarly, in recurrent cancer, PET-CT is somewhat limited in its ability to distinguish early postoperative inflammatory changes from tumor recurrence or persistence, but correlation with CT findings and the patient's history and examination helps to determine the significance and thus guide management.

The utility of FDG-PET imaging for patients with gynecologic cancers is an ongoing process. Most of the work in this area has been performed in patients with cervical and ovarian cancer [1]. FDG-PET is a quite accurate method for the detection of lymph node invasion with a sensitivity and specificity of 100% and 99.6%, respectively, in the detection of affected lymph nodes greater than 5 mm [1]. These values decrease in nodes smaller than 5 mm.

## 3. Diffusion-Weighted MRI (DWI)

MRI is the imagine technique with highest contrast and anatomic resolution, being the modality of choice for morphological evaluation of female pelvis disease. A new emerging functional technique that is currently finding a role in cancer imaging is DWI, which provides information about tissue cellularity and integrity of cellular membranes. DWI can be performed on most modern MRI machines with relative ease employing short exploration time (about 3 or 4 minutes) and does not need for contrast medium administration.

**Figure 1:**
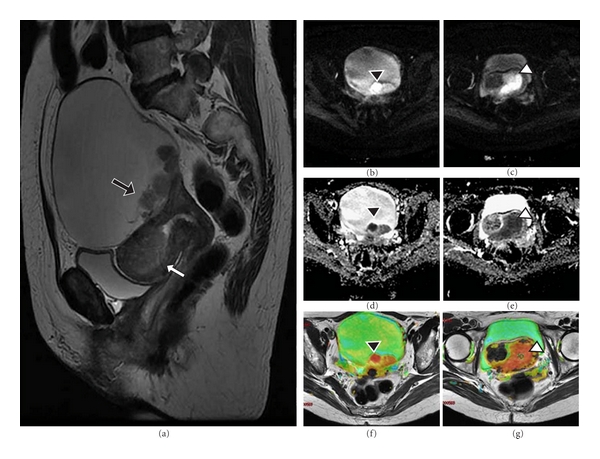
Synchronic ovarian serous cystadenocarcinoma and endometrium adenocarcinoma (stage IC) in a 60-year-old woman. (a) *Sagittal T2-WI image* shows a unilocular cystic mass with solid mural nodules (black arrow) of heterogeneous hyperintensity. There is an intermediate signal intensity mass filling the endometrial cavity (white arrow). ((b) and (c)) *DWI*: show high signal intensity corresponding to mural nodules (black arrowhead) and an ill-defined slightly hyperintense mass in the endometrial area (white arrowhead). ((d) and (e)) *ADC maps*: depict low signal intensity in the solid mural nodules (black arrowhead), with an ADC value of 0.81 × 10^−3^ mm^2^/s, while the cystic component presents values of 1.3 × 10^−3^ mm^2^/s. ADC value within the endometrial mass 0.79 × 10^−3^ mm^2^/s (white arrowhead). ((f) and (g)): Postprocessed axial images, in which fusion between DWI and T2-WI is obtained. Low ADC areas are represented in red.

At a fundamental level, DWI provides information on the random (Brownian) motion of water molecules in tissues. By comparing differences in the apparent diffusion between tissues, tissue characterization becomes possible. For example, a tumor would exhibit a more restricted diffusion if compared with a cyst because intact cellular membranes in a tumor would hinder the free movement of water molecules [4]. Diffusion is quantified by a parameter, the apparent diffusion coefficient (ADC; unit mm^2^/s), which is usually presented as a quantitative parametric map as gray-scale images. On ADC maps, tumors usually demonstrate low ADC values and appear as low signal intensity area compared with normal tissue (Figure 1). The quantitative ADC values can aid in lesion characterization, and can also be applied to evaluate treatment response of tumors [5].

 There are some pitfalls in the use of DWI and ADC values. In malignant tumors with low cellularity (e.g., well-differentiated adenocarcinomas or ovarian cancers with large cystic components), or in poorly differentiated necrotic tumors, restriction to water diffusion is likely to be much more limited and may not be visible at DWI [5]. Because of that, DWI is not useful for characterization of ovarian lesions.

False positives can also occur. Normal endometrium and other normal structures as reactive lymph nodes and bowel mucosa with high cellular density are also hyperintense on DWI. However, quantitative discrimination between normal endometrium and cancer is most often possible due to the significantly lower ADC value of the tumor.

Another limitation of DWI is the presence of suppression of background body signals, resulting in a lack of sufficient anatomical information. Readers should also be aware of the concept of T2 shine-through. This refers to hyperintensity on T2-WI influencing the DWI, which is seen as high signal on DWI, along with a bright area on the ADC map. It highlights the need to assess the DWI in correlation with the ADC map [5]. Due to these factors, ADC maps and DWI should never be interpreted separately, but together with anatomic images according to Figure 1. Fusion imaging between DWI and T2-WI in patients with gynecological cancer is able to depict both malignant tumors and anatomical information.

Because DWI is an emerging technique, there are few studies on the utility of DWI for gynecological imaging. Thus, further prospective study using larger numbers of patients and long-term followup is needed to establish the potential ability of DWI for gynecological diseases. Besides, there is a lack of standardization regarding the use of DWI in the assessment of gynecological tumors, including differences in the ADC values of similar diseases reported because of the use of different techniques [4]. Clearly, future standardization of protocols for both image acquisition and data analysis across imaging platforms is an important item.

## 4. Dynamic Contrast-Enhanced- (DCE-) MRI

DCE-MRI involves the acquisition of sequential images during the passage of a contrast agent through a particular tissue of interest. Dynamic imaging can depict the distribution of this agent by measuring variations in vessel and tissue enhancement over time. Moreover, the intensity of the enhancement has been shown to be related to the vascular density within tissue, while the rate and wash-out of enhancement is related to angiogenic factors such as microvessel density (MVD) and vascular endothelial growth factor (VEGF) [6–8]. Variations in contrast enhancement are associated with specific histopathological features of the tumor [9], with more aggressive tumors commonly exhibiting a more rapid and intense enhancement and washout, representing a higher vascular density and strong expression of VEGF. Therefore DCE-MRI has the ability to noninvasively characterize tissue vasculature including the antiangiogenic response of tumor tissue during therapeutic intervention. By providing additional insight into tumor perfusion and capillary permeability, this technique allows evaluation of treatment response more readily than delayed assessments of tumor size [10].

In gynecological oncology, DCE-MRI has been mostly evaluated in cervical tumors [11, 12]. There are two instances in which DCE-MRI may prove useful. First, it may improve detection of small tumors with a depth of stromal invasion between 3 and 5 mm, demonstrating a reported sensitivity of 92% compared with 23% with T2-WI [11]. Small tumors avidly enhance in the early dynamic phase as compared to the slight enhancement of the cervical epithelium and stroma. Second, it may help in distinguishing between recurrent tumors and radiation fibrosis [12]. It also has been studied in the characterization of malignancy of ovarian lesions [13].

## 5. Ovarian Cancer

In the characterization of an ovarian lesion, cost-benefit studies and net cost analysis have shown that the use of MR imaging in the evaluation of sonographically indeterminate adnexal lesions resulted in fewer surgical procedures, better patient triage, and net cost savings [14].

Although signal intensity characteristics can be used to narrow the differential diagnosis of an adnexal mass, no MR specific signal characteristics for ovarian cancer are recognized. Distinction of malignant from benign lesions is mainly based on morphologic criteria (Figure 1). The presence of papillary projections in a cystic mass is highly suggestive of ovarian cancer. Other features suggesting malignant etiology include necrosis, vascular septations thicker than 3 mm, septal nodularity, and single or multiple enhancing solid components within a cystic mass [15]. In patients with clinically or sonographically detected complex adnexal masses, MR imaging was shown to have 91% accuracy for the diagnosis of malignancy. Ancillary findings, such as ascites, peritoneal disease, or adenopathy, were the most significantly factors indicative of malignancy.

Functional imaging has been investigated for the detection and characterization of primary ovarian masses. Although FDG avid ovarian lesions in postmenopausal women are considered suspicious for malignancy, PET-CT is not recommended for primary cancer detection because of high false-positive rates. Physiologic ovarian uptake of FDG during different phases of the menstrual cycle [1] may be a limitation for detection of ovarian cancer. In addiction a variety of benign lesions, such as serous and mucinous cystadenomas, corpus luteum cysts, and dermoid cysts, are known to accumulate FDG and may contribute to false-positive results. Therefore, differentiating benign from malignant lesions using PET scans alone is impossible. The distinction generally requires correlation with a detailed clinical history and morphologic imaging such as ultrasound, CT, or MRI [16].

There are also several reports in literature about the clinical application of DWI to characterize ovarian tumors [17–19]. These studies show that, although ADC may help to differentiate between normal and cancerous tissue in the uterine cervix and endometrium (Figure 1), its usefulness may be limited in the case of ovarian lesions, a phenomenon attributable to their morphologic variety. Ovarian cysts containing blood (endometrial cysts), fat (teratomas), or pus (abscesses) present with higher water restriction and lower ADC values lower than some malignant ovarian cystic lesions. DCE-MRI can be useful in characterization of internal architecture of cystic lesions improving the detection of solid components [20]. DCE-MRI has also been shown to correlate with tumor angiogenesis biomarkers in ovarian cancer [13].

Regarding staging, ovarian cancer was traditionally staged on the basis of surgery and pathologic confirmation. Surgical staging is based on the International Federation of Obstetrics and Gynecology (FIGO) classification system.

CT and MRI were found to be highly accurate in the detection of inoperable tumor and the prediction of suboptimal debulking, being the two modalities equally effective in the detection of inoperable tumor [21]. Although CT is the primary imaging modality for staging ovarian cancer, a Radiologic Diagnostic Oncology Group study showed that MRI may be equal or superior to CT [15]. One advantage of MRI is that it provides better soft tissue contrast than does CT. Implants measuring 1 cm or less are difficult to detect by CT, decreasing the sensitivity to less of 50% for such small-volume disease [22].

**Figure 2:**
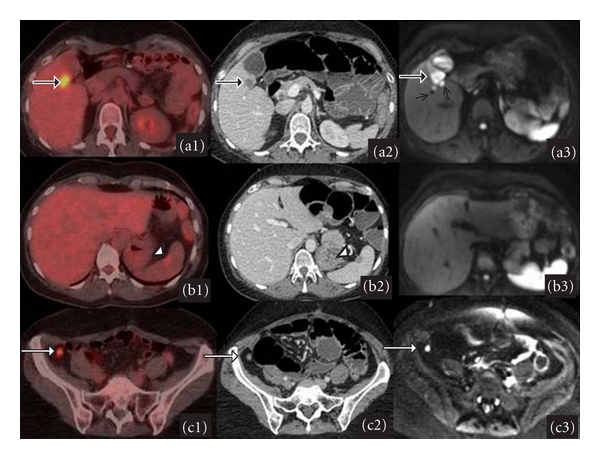
Peritoneal dissemination from recurrent ovarian serous adenocarcinoma in a 45-year-old woman. Image illustrates the diagnostic accuracy of different techniques. *First row *(a1)* PET*, (a2)* CT*,* and *(a3)* DWI.* Tumor deposit along the fissure of the hepatoduodenal ligament (arrows). PET and CT show only one lesion, being difficult to differentiate between a hepatic deposit and peritoneal implant. At DWI, two adjacent millimetric lesions are depicted, suggesting peritoneal implants. *Second row *(b1)* PET*, (b2)* CT*,* and *(b3)* DWI.* Implant in splenic helium, clearly visible in PET, but hardly visible at CT. However, DWI cannot detect the lesion because of the physiological hyperintensity of spleen. *Third row *(c1)* PET*, (c2)* CT and *(c3)* DWI.* A small implant in right paracolic gutter, measuring as small as 3 mm. DWI clearly demonstrates peritoneal disseminated implants as markedly hyperintense foci.

An important issue for staging ovarian cancer is the differentiation between stage III (liver surface implants) and stage IV (hepatic parenchymal metastases) disease with a direct impact on patient management (Figure 2). Sagittal or coronal reformatted images in multislice CT or MRI may assist in differentiating these two types. In the evaluation of liver parenchymal metastases CT and MRI perform similarly; however, MRI may be superior in the diagnosis of liver lesions in a reexisting liver disease setting [15].

The role of functional imaging techniques has been also explored for staging ovarian cancer. As previously mentioned, FDG-PET is very accurate for detection and localization of lesions to establish metastatic sites. Positive predictive value was 93% for peritoneal disease measuring equal or more than 5 mm [3, 23] (Figure 2).

DWI clearly discriminates the abnormal signal intensity of peritoneal dissemination from the signal arising from surrounding organs such as the bowel. Fujii et al. [24] showed that DWI was highly sensitive (90%) and specific (95.5%) for the evaluation of peritoneal dissemination and was of equal value as contrast-enhanced imaging in gynecological malignancy (Figure 2).

The followup of patients treated for ovarian cancer is usually performed with serial measurements of CA-125 and either CT scan or MRI of the abdomen and pelvis. It has been shown that in patients with a complete response to therapy, three consecutive elevations in CA-125 values are associated with a significant risk of recurrence and may occur before conventional imaging findings become positive [25].

As well as for staging, the functional techniques are very useful in the assessment of recurrence, although most studies have been conducted with FDG-PET. Second-look laparotomy (SLL) is the most accurate way of assessing the presence of microscopic and macroscopic disease; however, it is an invasive procedure. The use of FDG-PET imaging instead of SLL is reported to be feasible and cost effective [26]. FDG-PET helps localize the disease sites so that surgery or biopsy can be better directed. This is useful in cases where conventional imaging fails to detect recurrent disease [27]. FDG-PET may be more useful in detecting recurrence in the setting of negative conventional imaging studies and an increasing CA-125 [28]. In patients with an asymptomatic increase of CA-125, PET has a sensitivity of 87.5%. The combined sensitivity of PET and CA-125 is as high as 97.8%.

Another important issue of functional imaging and specifically PET-CT include the assessment of treatment response and prediction of treatment outcome. Avril et al. [29] proved that sequential FDG-PET predicted patient outcome as early as after the first cycle of neoadjuvant chemotherapy, being more accurate than clinical or histopathologic response criteria, including changes in CA-125 values.

Overall, FDG-PET [23, 26–29] and now DWI [24] hold promise in the evaluation of recurrent/residual disease and in assessment of treatment response where other radiographic findings are equivocal and uncertain. Newer tracers are being evaluated to improve detection; however, the sensitivity to microscopic disease is still limited.

## 6. Cervical Cancer

Staging of cervical cancer is based on clinical FIGO criteria. The major limitations of clinical staging are in the assessment of parametrial and pelvic sidewall invasion, the estimation of tumor size (especially if the tumor is primarily endocervical in location), and the evaluation of lymph node and distant metastases. The accurate pretreatment evaluation of these features is not only important for prognosis but also for determining the appropriate mode of treatment. Evidence shows that cross-sectional imaging is superior to clinical staging [30].

**Figure 3:**
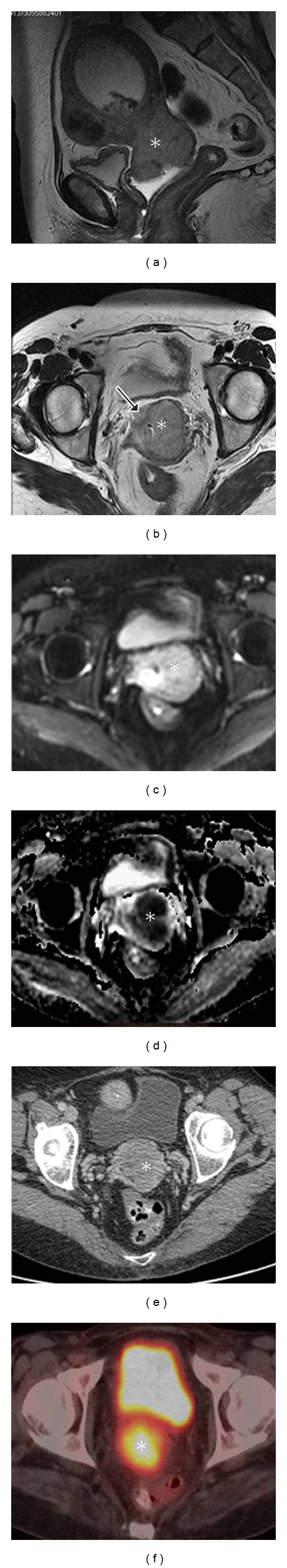
Stage IIb squamous cell carcinoma of the uterine cervix in a 38-year-old woman. (a)* Sagittal and *(b)* axial T2-WI *images of the uterus show a barrel-shaped cervical tumor (asterisks). Due to high anatomic resolution of MRI, axial T2-WI demonstrates disruption of the low signal cervical stromal ring and tumoral invasion of the right parametrium (black arrow). (c) *DWI*: shows a well-defined hyperintense mass in the cervical area. (d) *On the ADC map* the tumor is hypointense and shows ADC values as low as 0.79 × 10^−3^ mm^2^/s. (e) *Axial postcontrast CT image* hardly depicts cervical cancer, and is not a reliable tool for distinguishing tumor infiltration of adjacent parametrial structures. (f) *PET-CT*: the tumor is clearly visible because of its high metabolic activity, but it is not possible to distinguish parametrial infiltration.

Owing to its superior soft tissue delineation and multiplanar capability, MRI is considered the most accurate imaging modality for the evaluation of cervical cancer, and is now an integral part of local staging for patients with cervical cancer. The overall staging accuracy of MRI ranges from 77 to 90% [30, 31] (Figure 3). MRI is superior to clinical evaluation in the assessment of tumor size and provides measurements comparable to surgical measurements in most cases [30]. The reported accuracy of MRI in the detection of parametrial invasion ranges from 77% to 96% [30]. Because of its excellent soft-tissue resolution and the use of endovaginal gel, MRI is advantageous in the depiction of vaginal involvement and rectal and bladder invasion.

**Figure 4:**
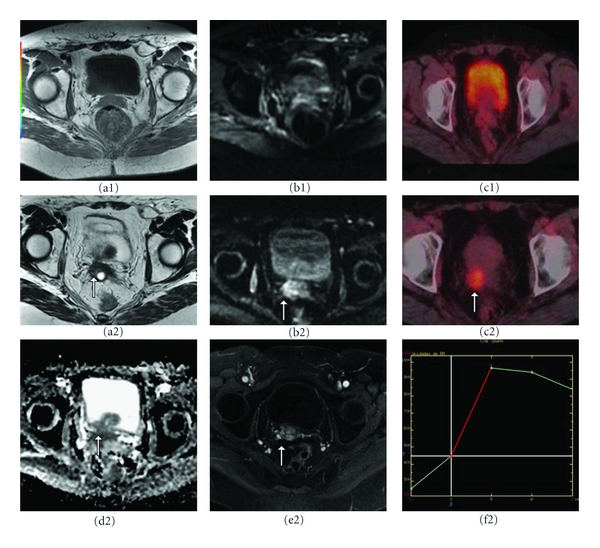
Proven vaginal fornix recurrence after radical surgery for cervical cancer in a 65-year-old woman. *First row *(a1)* axial T1-WI*, (b1)* DWI and *(c1)* PET-CT images* show no remarkable alteration except for an abnormal signal at DWI in the right vaginal fornix (white arrow), with an abnormal ADC value, measuring 1.3 × 10^−3^ mm^2^/s. *Second and Third rows*: six months later (a2)* axial T2-WI*, (b2)* DWI*, (c2)* PET-CT*,* and *(d2)* ADC map show that* the ADC value in the right vaginal fornix had decreased to 1.1 × 10^−3^ mm^2^/s, corresponding to a hypermetabolic focus at PET-CT (white arrow). (e2)* and *(f2)* DCE-MRI* depicted a small focus with faster first-pass contrast agent enhancement, with a type III kinetic curve (washout type), which is indicative of malignancy.

Although cervical cancer demonstrates variable contrast enhancement, DCE-MRI may improve assessment of small tumors (Figure 4). Postcontrast MRI may also help in the detection of bladder or rectal wall invasion or delineation of fistulas.

**Figure 5:**
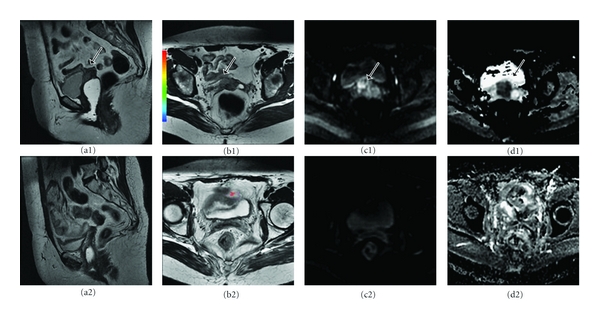
Central recurrence after radical surgery for cervical cancer in a 65-year-old woman. *First row *(a1)* and *(b1)* axial and sagittal T2-WI* of the uterus show a soft-tissue mass of high signal intensity in the vaginal vault. (c1)* DWI* shows a well-defined hyperintense area corresponding to the mass. (d1)* on ADC map *the tumor is hypointense (arrow). The ADC value within the mass is 0.87 × 10^−3^ mm^2^/s. *Second row* six months after radiation treatment, *sagittal and axial T2-WI *(a2)* and *(b2) show resolution of the mass. (c2)* and *(d2)* DWI and ADC map* showed no signal intensity alteration, with an ADC value of 1.4 × 10^−3^ mm^2^/s.

Functional information from PET and now with DWI and DCE-MRI can supplement morphologic information obtained with conventional cross-sectional imaging methods (Figures 3, 4, and 5). Although the current use of these techniques in the initial evaluation of cervical cancer is still under investigation, PET, DWI, and DCE-MRI are an effective adjunct to CT and MR imaging in evaluating lymph node involvement, distant metastases, and treatment response.

The use of FDG-PET is now well established in cervical cancer, since most cervical tumors are FDG avid (Figure 3). Adenocarcinomas, which usually have a low FDG uptake, are an exception [2]. PET-CT can be used at the time of presentation for staging and prognostic evaluation, to monitor response, to detect recurrence, and to plan radiotherapy.

In the context of primary tumor staging, PET-CT plays a valuable role in the evaluation of lymph node metastases. Nodal metastases are frequent in patients with advanced disease (i.e., FIGO stages IIB to IVB) and FDG-PET has been demonstrated to have a high specificity for the detection of nodes in this group of patients [32]. FDG-PET also improves initial staging in cases of advanced disease by demonstrating unexpected sites of disease beyond the pelvis or retroperitoneum, such as supraclavicular nodal metastases [32]. By contrast, the value of FDG-PET in early stage disease (i.e., FIGO stages I to IIA) is questionable. Many studies have reported low sensitivities for the detection of nodal metastases, ranging from 25 to 73% [33].

As mentioned above DWI are readily usable in pelvis, adding the possibility of discriminating between benign and malignant lesions of the uterus. Cervical cancer has shown to have significantly lower ADC values as compared to normal cervical tissue [34–36]. According to Tamai et al. [35] the average median ADC of cervical cancers was significantly lower than normal cervix (1.09 × 10^−3^ versus 2.09 × 10^−3^ mm^2^/s (Figure 2)). In opposition to PET/CT, high-grade adenocarcinomas typically have high cellular density and so would be expected to have lower ADC values [5].

The appropriate long-term clinical surveillance protocol for patients with advanced cervical cancer is poorly defined. It is imperative to accurately identify those patients deemed suitable for radical steps such as pelvic exenteration, which is associated with considerable morbidity rates. Up to a third of women who elected to undergo exenteration were deemed unsuitable at the time of surgery, owing to the fact that the extent of disease was more advanced than was despicted in the preoperation workup [37]. On this regard, combining functional techniques on top of conventional ones allows detection of small volumes of active tumor, improving a better presurgical patient selection of candidates to pelvic exenteration.

After chemoradiation treatment, two important issues are, in the first place, to distinguish postradiation changes from recurrent tumor, and in the second place, to assess treatment response. T2-WI MRI has high sensitivity (90%-91%) but low specificity (22%–38%) for recurrent disease in the cervix [38]. This low specificity is caused by the fact that benign conditions such as edema, inflammation, and necrosis also may cause increased T2 signal, therefore mimicking residual tumor.

In the context of recurrence, the applications of PET-CT include identifying residual/recurrent disease at the primary site, assessment of nodal disease, detection of distant metastases, and radiotherapy field planning [39]. A study found that the sensitivity of PET for detecting recurrence was 80% in asymptomatic women and 100% in symptomatic women [40]. FDG-PET also can be useful in women who present with elevated markers but negative conventional imaging, like in ovarian tumors.

PET-CT has also been proved as biomarker of tumor response. It has been demonstrated to have a strong association between metabolic response and patient outcome. Grigsby et al. [41] used pretreatment FDG-PET to evaluated tumoral response in patients who had received nonsurgical treatment, with higher survival rates in patients who demonstrated no residual FDG activity. FDG-PET/CT can be utilized to outline the irradiation targets for the FDG-avid lesions [42], and for targeting the brachytherapy portion of the irradiation treatment [43].

DCE-MRI has been investigated in various studies as an early indicator of tumor response to therapy and as a tool for detecting small recurrences (Figure 4). The combination of DCE-MRI with T2-WI improved specificity from 38% to 67% [38]. DCE-MRI parameters such as relative signal intensity or peak enhancement have been investigated as predictive markers of response even prior to the start of a treatment regimen while changes in enhancement and signal intensity in very early stages of therapy have been shown to be associated with improved local tumor control [44].

DWI is now being studied in these issues. Because many therapies induce cellular lysis, an increase in water diffusion distances within tumors is expected, with increasing ADC values following successful therapy. Naganawa et al. [45] found significant increases in lesion ADC values in patients with cervical cancer who were treated with combined chemoradiation therapy. The success of therapy can be assessed both quantitatively with ADC measurements and qualitatively by inspecting signal intensity on DWI (Figure 5).

Rapid increases in ADC values are seen following chemotherapy whereas changes in perfusion as depicted with dynamic sequences have a later onset, usually occurring after one to two cycles of chemotherapy. Radiation therapy may also cause initially increased ADC values due to hyperemia. Therefore, DWI and DCE-MRI are complementary in assessing response to therapy and should be interpreted with full knowledge of the patient's treatment schedule. In the same way, hyperintense signal on DWI associated with lower ADC values is suggestive of an active tumor [46]. Although to date there are no publications evaluating the role of DWI in tumoral detection; in our experience monitoring ADC values is very useful, allowing detecting small recurrences (Figure 4).

## 7. Endometrial Cancer

Similar to cervical cancer, staging of endometrial cancer is based on surgicopathologic FIGO criteria. Surgical staging, however, is not suitable for women who are not good surgical candidates because of older age, obesity, and other medical comorbidities.

FDG-PET has been utilized in the pretreatment evaluation of patients with endometrial cancer. The application of FDG-PET/CT has been reported to improve detection of pelvic nodal and/or soft-tissue metastases and extrapelvic metastases [47], but little is addressed in assessing myometrial invasion [48]. Also in premenopausal women, the uterus will often demonstrate FDG uptake most commonly during the menstrual flow [1], not being possible to differentiate tumoral from physiologic endometrial uptake, even with the help of CT.

MRI is the most accurate modality for the pretreatment evaluation of endometrial cancer. The overall staging accuracy of conventional MR imaging was reported to be 83% to 92%, with 87% of sensitivity and 91% of specificity in assessing myometrial infiltration, 80% of sensitivity and 96% of specificity for cervical invasion, and 50% of sensitivity and 95% of specificity for lymph node assessment [49]. Like all other cross-sectional imaging methods, MR imaging is limited in the assessment of lymph node status because it does not allow clear differentiation between metastatic and nonmetastatic lymph nodes of similar size. In this particular issue FDG-PET is clearly superior.

Recent developments in functional techniques have magnified the role and potential of MRI, especially in determining the presence of myometrial invasion. Determining the presence of myometrial invasion is a critical factor, because the presence of deep myometrial invasion is associated to six- to seven-fold increased prevalence of lymph node metastases, as compared to patients with absent or lower than 50% myometrial invasion [50]. Preoperative determination of myometrial invasion is of great help in determining lymphadenectomy extent.

DCE-MRI has traditionally been used because T2WI may not always appreciate the junctional zone required for determining depth of invasion, particularly in postmenopausal patients or in patients with myometrial thinning. DCE-MRI is especially valuable in demonstrating myometrial invasion because the majority of tumors are hypovascular relative to the vascular myometrium. However, a significant number of tumors are either iso- or hypervascular relative to the myometrium.

**Figure 6:**
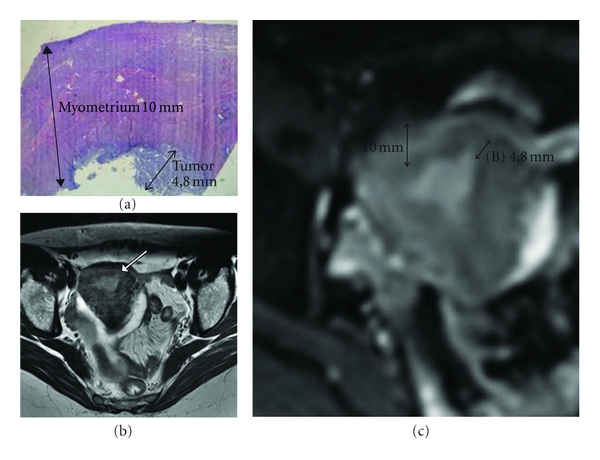
Stage Ib endometrial cancer in a 50-year-old woman. (a)* Axial T2-WI*. The invasion ratio was tumor invasion depth (b) divided by myometrial thickness (A + B) measured at *DWI *(b) and (a)* histopathologic examination* (hematoxylin-eosin stain).

On this regard, because DWI is essentially independent of differences in vascularity, it is useful for determining T stage in such cases. Shen et al. [51] also found that DWI depicted tumor foci that were not appreciated with T2-WI or dynamic sequences, such as elsewhere in the uterus or in peritoneal spread. In addition, it may not always be possible to perform contrast-enhanced imaging due to factors such as renal failure. Lin et al. [52] found that fused T2-WI and DWI at 3 T have potential in the assessment of myometrial invasion in a noninvasive manner, with an excellent interreader agreement and a diagnostic performance as high as that of DCE-MRI (Figure 6).

Similar to cervical cancer, the possibility of discriminating between benign and malignant lesions of the uterus with DWI has been investigated. The ADC range of values of endometrial cancer (0.88–0.98 × 10^−3^ mm^2^/s) is significantly lower than that of endometrial polyps (1.27–1.58 × 10^−3^ mm^2^/s) or normal endometrium (1.53 × 10^−3^ mm^2^/s) [51, 53]. DWI should be considered as part of routine preoperative MRI evaluation for endometrial cancer, but further study using larger numbers of patients and long-term followup is needed to establish the accuracy of ADC measurement for endometrial cancer.

## 8. Conclusion

The utility of FDG-PET/CT imaging for patients with gynecologic cancers is an ongoing process. FDG PET/CT can significantly assess the extent of primary and recurrent cancer and, hence, often alters patient management. It has high accuracy especially in the detection of nodal metastasis, peritoneal implants less than 10 mm, and small local recurrences. Nevertheless, because FDG PET/CT has less contrast and anatomic resolution than MRI, it cannot replace MRI in local preoperative staging.

DWI, a new functional MRI technique, achieves image contrast by evaluating the random motion of water molecules within tissues. It has many advantages: the additional scan time is relatively short and intravenous contrast is not needed, so that it can be applied to patients with renal impairment and integrated into routine scanning protocols. Potential applications of fMRI and specifically DWI include the challenging topics of distinguishing tumor from nontumor tissue in cervical and endometrial cancer, presurgical mapping, assessment of treatment response and prediction of treatment outcome. However, DWI has a minor role in detecting nodal metastasis.

Because DWI is an emerging technique, there are only few studies on its applications on gynecological imaging. In our experience, the combination of DWI with anatomic imaging increases the diagnostic accuracy in oncologic patients. However, further prospective study using larger numbers of patients and long-term followup are needed to establish the potential ability of DWI for gynecological diseases.

Both PET-CT and now fMRI have proved to be very valuable tools for the evaluation of gynecologic tumors. Most papers try to compare these techniques, but in our experience they both are complementary in the management of these patients. Meanwhile PET-CT is superior in the diagnosis of ganglionar disease; fMRI presents higher accuracy in local preoperative staging and distinguishing early postradiation changes from recurrent tumor. Both techniques can be used as biomarkers of tumor response and present high accuracy in the diagnosis of local recurrence and peritoneal dissemination, with complementary roles depending on histological type, anatomic location, and tumoral volume.

##  Conflict of Interests 

The authors have no conflict of interest to declare.

##  Author's Contributions

M. J. de la Peña collaborated on the original idea and revision of the paper. R. C. Alonso collaborated in drafting the paper.
